# Cofactors in allergic reactions to food: physical exercise and alcohol are the most important

**DOI:** 10.1002/iid3.120

**Published:** 2016-09-15

**Authors:** Astrid Versluis, Harmieke van Os‐Medendorp, Astrid G. Kruizinga, W. Marty Blom, Geert F. Houben, André C. Knulst

**Affiliations:** ^1^ UMC UtrechtUtrechtThe Netherlands; ^2^ TNOZeistThe Netherlands

**Keywords:** cofactor, exercise, food allergy

## Abstract

**Introduction:**

Involvement of cofactors, like physical exercise, alcohol consumption and use of several types of medication, are associated with more severe food allergic symptoms. However, there is limited evidence on how often cofactors play a role in food allergic reactions. The study aimed to get more insight into the frequency of exposure to cofactors and how often cofactors are associated with more severe symptoms in food allergic patients.

**Methods:**

A questionnaire was completed by patients visiting the Allergology outpatient clinic. Patients with food allergy were included. Outcome measures were the frequency of medication use of medication groups that might act as cofactor and the frequency that physical exercise, alcohol consumption and use of analgesics are associated with more severe food allergic symptoms.

**Results:**

Four hundred ninety‐six patients were included in the study. The frequency with which patients used one or more types of medication that might act as cofactors was 7.7%: antacids/acid neutralizing medication (5%), NSAIDs (2%), beta blockers (0.6%), angiotensin‐converting enzyme inhibitors (0.6%), and angiotensin receptor blockers (0.2%). Of all patients, 13% reported more severe symptoms to food after involvement of one or more of the cofactors: physical exercise (10%), alcohol consumption (5%), and use of analgesics (0.6%). Sixty‐five percent did not know if these cofactors caused more severe symptoms; 22% reported that these cofactors had no effect.

**Conclusions:**

Only a small percentage of patients (7.7%) used medication that might aggravate food allergic reactions. Physical exercise and alcohol consumption were the most frequently reported cofactors, but occurring still in only 10% or less.

## Introduction

Food allergy is an important health problem. The point prevalence of food allergy is estimated to affect around 1–3% of the European population, assessed by clinical history and IgE and/or food challenge 1. Most food allergic patients are confronted with unexpected allergic reactions despite their avoidance diet 2. Managing avoidance of allergenic food to prevent allergic reactions places a psychological burden on patients and has a negative impact on quality of life 3, 4. To help patients in managing their diets, European Union regulations prescribe that the fourteen most frequently used ingredients that can cause hypersensitivity or intolerance must be listed on food labels 5.

It is reported that in some patients with food allergy, allergic reactions are more severe if a cofactor is involved 6, 7. EAACI guidelines 8 define cofactors as patient‐related or external circumstances that are associated with more severe allergic reactions. Cofactors are in literature also referred to as augmentation‐, additional or associated factors 6, 7, 9. In this study the term cofactor is defined as external circumstances that are associated with more severe allergic symptoms.

Cofactors, such as alcohol, physical exercise, infections and use of some types of medication (e.g., nonsteroidal anti‐inflammatory drugs (NSAIDs), antacids, acid neutralizing medication, beta blockers, angiotensin receptor blockers (ARBs) and angiotensin‐converting enzyme inhibitors (ACEIs)) might influence the occurrence of allergic reactions 6, 7, 10, 11. Literature reports that cofactors are involved in 25.6–39% of the anaphylactic reactions to food in adults 10, 12. The underlying mechanisms of cofactor in augmenting food allergic reactions are hardly understood. One suggested mechanism is an increased gastrointestinal absorption of protein, caused by underlying processes like gastrointestinal hyperpermeability after physical exercise or intake of NSAID's, or relaxation of tight junctions in gut epithelium after intake of alcohol 6, 7, 13, 14, 15. For intake of alcohol other mechanisms are suggested as well: (1) alcohol has a direct influence on total IgE levels, which is related to the amount of consumption 7, 16, 17; (2) some patients probably react to ingredients of alcoholic beverages 7. Other mechanisms suggested with respect to physical exercise are: (1) increased blood circulation leading to increased influx of allergen in the gut 7; (2) basophil activation and increased histamine releasability through lowered pH and increased osmolarity 6, 7, 13; (3) elevated IL‐6 upregulates tissue transglutaminase (tTG) enzymes, resulting in peptide aggregation which leads to increased IgE cross‐linking 6, 7, 13; and (4) redistribution of blood that transports the allergen from the gut to skeletal muscle and/or skin where phenotypically different mast cells reside, resulting in an altered mediator release 13.

The available evidence on the frequency of involvement of cofactors and the influence on food allergic reactions is scarce. Besides, most studies have been conducted in patients with severe allergic reactions 10, 12, 18, 19. Further, the results of different studies are not consistent in frequency of involvement of cofactors in food allergic reactions. More evidence on the role of cofactors is important for diagnostics and doctors’ advice to patients and on population level to help the food industry and regulatory authorities to design appropriate food safety strategies 6, 7, 20. The aim of this study was to get more insight into the frequency of exposure to cofactors and how often cofactors are associated with more severe symptoms to food in patients with a doctor diagnosed food allergy.

## Methods

### Study design, setting, data collection, and participants

This was a database study. Patients referred to Allergology outpatient clinic of the University Medical Center Utrecht (UMCU) because of a suspected food allergy, were asked to fill in a one‐time questionnaire before the first consultation. The questionnaire consisted of topics about food allergy, atopic comorbidities, medication use and if physical exercise, alcohol consumption and use of analgesics within 2 h after consumption of the suspected food causes more severe symptoms. The results of this questionnaire and conducted diagnostic tests (skin prick tests [SPT], ImmunoCAP and food challenges) were collected in databases between November 2002 and August 2012.

The study population consisted of patients ≥16 years of age with a food allergy. The food allergy diagnosis was established based on patient reported allergic symptoms to food and a positive SPT or ImmunoCAP (conducted within a year before or after the reported symptoms) or food challenge for the same type of food. An exclusion criterion was inability to read or write the Dutch language.

### Outcome measures and patient demographics

The first outcome measure was the frequency that patients reported an association between physical exercise, alcohol consumption or use of analgesics with increased severity of allergic symptoms to food.

The second outcome measure was the frequency of medication use from medication groups that are suggested in literature as cofactors; namely antacids/acid neutralizing medication, NSAIDs, beta blockers, ARBs, and ACEIs 6, 7.

Patient characteristics comprised gender, age, atopic comorbidities (asthma, allergic rhino conjunctivitis and atopic dermatitis), type and severity of food allergy, the mean number of different food allergies and use of medication that could suppress allergic symptoms (systemic corticosteroids, immunosuppressive drugs, antihistamines, inhaled betamimetics, and inhaled corticosteroids). Severity of food allergy was classified according to an adapted version of the Mueller allergy severity grading scale. Reactions with local oral symptoms were classified as Mueller 0; with skin and mucosal symptoms as Mueller 1; with gastrointestinal symptoms as Mueller 2; with respiratory symptoms as Muller 3 and cardiovascular symptoms as Mueller 4 21, 22. The different types of food allergy were divided into the fourteen major food allergies and to fruit, vegetables and other types of food allergy 5. A pulmonologist and dermatologist were consulted to diagnose asthma, allergic rhino conjunctivitis and atopic dermatitis based on the available patients’ data and international guidelines 23, 24, 25. Patients were considered asthmatic if they (ever) had two or more respiratory complaints (dyspnea, coughing and/or wheezing). Patients were considered to have allergic rhino conjunctivitis if they (ever) had eye‐ and/or nose complaints during a specific season or allergic symptoms to dogs or cats in combination with a positive sensitization (SPT or ImmunoCAP) to the corresponding aeroallergen. The tree/grass pollen season was set on the months January to August, mugwort season in August and September and dust mites season during the entire year. Patients were considered to have atopic dermatitis if they (ever) had pruritus in combination with two or more of the following criteria: (ever) had xerosis, involvement of classical locations (face/neck, elbow crease, and/or on the back of the knees) and personal history of asthma or allergic rhino conjunctivitis.

### Study size

In order to include a representative group of the available population of patients with food allergy visiting the Allergology outpatient clinic for the first time over a period of 10 years (estimated at 200 per year in the UMCU) the sample size of that group was calculated using the Raosoft Sample Size calculator 26. With a margin of error of 1%, a confidence level of 95% and a response rate of 50%, 499 new patients should be included.

### Statistical methods

IBM SPSS Statistics 21 (IBM Corporation, Armonk, NY) was used for data analysis. Descriptive statistics were used to analyze outcome data. Patient demographics on a categorical scale were analyzed by calculating frequency data (n/percentages) and on a ratio scale by calculating the mean and standard deviation. To analyze differences in the frequency of cofactors between patients with mild or more severe allergic symptoms the chi square test was used (or the Fisher's exact test in case of small numbers). The use of medication was clustered in groups (antacids/acid neutralizing medication, NSAIDs, beta blockers, ARBs, ACEIs, other types of medication). The frequency with which patients used medication of one of these groups was calculated (n/percentage). The chi‐square test was used to assess differences between subgroups of age (or the Fisher's exact test in case of small numbers). Because of the explorative design of the study, a *P* value <0.05 was considered statistically significant. Missing data was taken into account by coding them as missing and was excluded from analysis.

### Ethics

The local Medical Ethics Review Committee confirmed that the Medical Research Involving Human Patients Act (WMO) does not apply to the study (protocol number: 13‐520/C).

## Results

### Patient characteristics

Of the 1173 patients who filled in the questionnaire, 496 patients with a confirmed food allergy were included. In total, 677 patients were excluded because of not having food allergy (*n* = 671) or being <16 years of age (*n* = 6).

The mean age of the included patients was 33 years (SD 12.5). Most patients had allergy to several types of food (mean: 2.9 different foods). Of the major food allergies, the most common were hazelnut (43%) and peanut (38%). The severity of food allergy of patients varied from mild/moderate (Mueller 0–2) in 48% of the patients to severe (Mueller 3–4) in 52%. Of all patients, 88% had one or more atopic comorbidities: asthma (62%), atopic dermatitis (67%) and/or allergic rhino conjunctivitis (74%). Medication that could suppress allergic symptoms was used daily or on demand in 67% of the patients; systemic corticosteroids and/or immunosuppressive drugs (9%), antihistamines (56%), inhaled betamimetics (24%) and inhaled corticosteroids (22%) (Tables 1 and 2).

**Table 1:** Patient characteristics, atopic comorbidities and severity of the most severe food allergy

	*n* (%) (*n* = 496)
Gender: female (missing values: *n* = 3)	349 (70%)
Mean age in years (SD, min‐max) (missing values: *n* = 1)	33 (12.5, 16–79)
Atopic comorbidities	
Asthma, atopic dermatitis and/or allergic rhino conjunctivitis	436 (88%)
Asthma (missing values: *n* = 12)	302 (62%)
Atopic dermatitis (missing values: *n* = 52)	232 (67%)
Allergic rhino conjunctivitis (missing values: *n* = 12)	359 (74%)
Medication that could suppress allergic symptoms (on demand and daily use)	
Uses medication from ≥1 of the below medication groups	334 (67%)
Systemic corticosteroids and/or immunosuppressive drugs	46 (9%)
Antihistamines	276 (56%)
Inhaled betamimetics	118 (24%)
Inhaled corticosteroids	108 (22%)
Emergency medication prescribed for food allergy^1^	
Antihistamines	299 (60%)
Corticosteroids	72 (15%)
Adrenaline auto‐injector	154 (31%)
Emergency medication, type unknown	13 (3%)
No emergency medication	143 (29%)
Severity (Mueller) of the most severe food allergy	
Mueller 0	88 (18%)
Mueller I	86 (17%)
Mueller 2	64 (13%)
Mueller 3	194 (39%)
Mueller 4	64 (13%)

^1^ This data is reported by patients before the first consultation at the outpatient department Allergology.

**Table 2:** Distribution and mean number of food allergies

	*n* (%)	Positive CAPs	Positive SPTs	*n* (%)	*n* (%)
Distribution of food allergies	All patients (*n* = 496)	N (min‐max, median) (*n* = 496)	N (range wheal, median) (*n* = 496)	Mueller 0–2 (*n* = 238)	Mueller 3–4 (*n* = 258)
List of 10 out of 14 major food allergens^1^					
Cow's milk	34 (7%)	31 (0.40–101, 2.02)	11 (1–4, 2)	12 (5%)	22 (9%)
Hen's egg	29 (6%)	26 (0.40–101, 2.38)	11 (1–4, 2)	14 (6%)	15 (5%)
Peanut	191 (39%)	114 (0.39–101, 4.32)	136 (1–5, 2)	75 (32%)	116 (45%)
Tree nuts	283 (57%)				
Hazelnut	212 (43%)	127 (0.36–100, 6.64)	156 (1–4, 2)	93 (39%)	119 (46%)
Almond	125 (25%)	30 (0.4–5.70, 1.69)	105 (1–4, 2)	61 (26%)	64 (25%)
Walnut	115 (23%)	48 (0.4–45.10, 1.95)	84 (1–4, 2)	48 (20%)	67 (26%)
Brazil nut	9 (2%)	9 (0.73–42, 2.68)	Not determined	0	9 (4%)
Pistachio	24 (5%)	24 (0.4–100, 1.44)	Not determined	6 (3%)	18 (7%)
Cashew nut	41 (8%)	28 (0.39–57, 3.55)	23 (1–4, 2)	11 (5%)	30 (12%)
Fish^2^	4 (1%)			3 (1%)	1 (0.4%)
Cod		3 (0.5–10.90, 6.30)	2 (2–4, 3)		
Crustaceans^2^	29 (6%)			9 (4%)	20 (8%)
Shrimp		21 (0.4–61, 2.23)	16 (1–4, 2)		
Lobster		14 (0.36–55, 2.12)	5 (1–3, 2)		
Crab		13 (0.39–52, 1.70)	6 (1–3, 1)		
Sesame	33 (7%)	22 (0.5–82, 4.85)	22 (1–4, 2)	9 (4%)	24 (9%)
Soy	46 (9%)	25 (0.4–12.7, 1.14)	33 (1–4, 2)	14 (6%)	32 (12%)
Lupin	2 (0.4%)	2 (3.86–19.90, 11.88)	Not determined	1 (0.4%)	1 (0.4%)
Celery	36 (7%)	4 (0.5–4.87, 2.33)	34 (1–4, 2)	16 (7%)	20 (8%)
Other allergens, not belonging to the major allergens					
Fruit (all)^3^	338 (68%)			175 (74%)	163 (63%)
Fruit (top 3)					
Apple	270 (54%)	154 (0.38–58.10, 3.73)	215 (1–5, 3)		
Kiwi	171 (35%)	45 (0.36–31.10, 1.20)	147 (1–5, 3)		
Peach	124 (25%)	40 (0.44–70.20, 2.60)	110 (1–2, 2)		
Vegetables (all)^3^, ^4^	154 (31%)			72 (30%)	82 (32%)
Vegetables (top 3)					
Tomato	82 (17%)	20 (0.4–26.10, 1.55)	73 (1–4, 2)		
Carrot	73 (15%)	17 (0.82–45,2.78)	67 (1–4, 2)		
Paprika	44 (9%)	5 (0.40–3.53, 0.71)	41 (1–3, 2)		
Other food allergies	6 (1%)			1 (0.4%)	5 (2%)
Mean number of food allergies^3^ (SD, min‐max)	2.9 (1.87, 1–9)			2.6 (1.74, 1–9)	3.1 (1.95, 1–9)

^1^ Of the 14 major allergens sulphur dioxide, cereals containing gluten, molluscs and mustard were not specifically evaluated in the questionnaire. Of the tree nuts, pecan nut and macadamia nut were not specifically addressed in the questionnaire. The questionnaire contained an item “other food allergies.”

^2^ Allergy to any species.

^3^ Allergy to one or more fruits considered as 1 food allergy, allergy to one or more vegetable(s) considered as 1 food allergy and other food allergies considered as 1 food allergy.

^4^ Celery not included.

### Use of medication in the total food allergic population

Medication use was analyzed to gain insight into the frequency that patients used medication previously suggested in literature as a cofactor.

Of all patients, 7.7% (95% CI: 5–10%) used medication that might act as a cofactor. The most commonly used types of medication were antacids/acid neutralizing medication (5%) and NSAIDs (2.2%). Beta blockers, ACEIs, ARBs were used by ≤0.6% of the patients.

Patients above 21 years of age used significantly (*P* = 0.028) more frequent medication that could function as a cofactor (9%) compared with adolescents (16–21 years of age) (3%). There was no significant difference in use of medication from the individual medication groups between the two age‐groups, whereas beta blockers, ACEIs and ARBs were only used by patients above 21 years of age (Table 3).

**Table 3:** Frequency of medication use from medication groups that might act as a cofactors

Medication use	*n* (%) All patients (*n* = 496)	*n* (%) ≤21 years of age (*n* = 108)	*n* (%) >21 years of age (*n* = 387)	*P*‐value ≤21 years of age vs. >21 years of age (*n* = 495)
Uses medication from ≥1 of the below medication groups	38 (7.7%)	3 (3%)	35 (9%)	0.028
Antacids/acid neutralizing medication	25 (5.0%)	1 (0.9%)	24 (6%)	0.025
NSAIDs	11 (2.2%)	2 (1.9%)	9 (2.3%)	1.000
Beta blockers	3 (0.6%)	0	3 (0.8%)	1.000
Angiotensin‐converting enzyme inhibitors (ACEIs)	3 (0.6%)	0	3 (0.8%)	1.000
Angiotensin‐receptor blockers (ARBs)	1 (0.2%)	0	1 (0.3%)	1.000

### Frequency of physical exercise, alcohol consumption, and use of analgesics as cofactor in food allergic reactions

Of all patients, 13% (95% CI: 10–16%) reported experiencing more severe symptoms to food after involvement of one or more of the cofactors: physical exercise in 10%, alcohol consumption in 5% and use of analgesics in 0.6%. Of the patients reporting the cofactor physical exercise, one patient had FDEIA (to chicken meat and hen's egg). Sixty‐five percent of all patients reported that they did not know if involvement of one of these cofactors caused more severe symptoms. Twenty‐two percent of the patients reported that cofactors had no effect on their allergic symptoms.

Patients with mild or moderate food allergy (Mueller 0–2) reported significantly (*P* = 0.037) less frequently that involvement of cofactors caused more severe symptoms, in comparison with patients with severe food allergy (Mueller 3–4), resp. 10% versus 16%. There was no significant difference between patients with mild or moderate food allergy and patients with severe food allergy, with regard to the frequency of the involvement of physical exercise and use of analgesic, the frequencies were resp. 7% versus 12% and 0% versus 1%. The involvement of alcohol consumption causing more severe symptoms was reported in 5% of the patients in both groups.

The frequency of cofactors between adolescents (16–21 years of age) and adults above 21 years of age was resp. 16% versus 12%. Physical exercise was more frequently reported as a cofactor in adolescents (13%) in comparison with patients above 21 years of age (9%). Alcohol consumption and use of analgesics were reported less frequently in adolescents compared with adults above 21 years of age, resp. 3% versus 5% and 0% versus 1%. None of the differences were statistically significant (Table 4).

**Table 4:** Frequency of cofactors in all patients and subgroup analyses regarding severity of food allergy and age

Cofactors	*n* (%) All patients (*n* = 491)	*n* (%) Mueller 0–2 (*n* = 236)	*n* (%) Mueller 3–4 (*n* = 255)	*P*‐value Mueller 0–2 vs. Mueller 3–4 (*n* = 491)	*n* (%) ≤21 years of age (*n* = 108)	*n* (%) >21 years of age (*n* = 382)	*P*‐value ≤21 years of age vs. >21 years of age (*n* = 490)
Physical exercise, alcohol consumption and/or analgesic use	64 (13%)	23 (10%)	41 (16%)	0.037	17 (16%)	47 (12%)	0.349
Physical exercise	47 (10%)	17 (7%)	30 (12%)	0.086	14 (13%)	33 (9%)	0.178
Alcohol consumption	24 (5%)	12 (5%)	12 (5%)	0.846	3 (3%)	21 (5%)	0.248
Analgesic use	3 (0.6%)	0	3 (1%)	0.250	0	3 (1%)	1.000
Unknown to the patient	317 (65%)	156 (66%)	161 (63%)	0.493	62 (57%)	255 (67%)	0.073
No effect	110 (22%)	57 (24%)	53 (21%)	0.371	29 (27%)	80 (21%)	0.192

## Discussion

This study illustrates the presence and role of cofactors in patients with a doctor diagnosed food allergy. In this population, 7.7% of patients used medication that might act as a cofactor, whereof antacids/acid neutralizing medication and NSAIDs were most frequently used. This study further showed that 13% of the food allergic patients reported more severe allergic symptoms to food after involvement of one or more of the following cofactors: physical exercise (10%), alcohol consumption (5%) and use of analgesics (0.6%). More than half of the patients (65%) indicated not to have known if one of these cofactors had been associated with their allergic symptoms to food.

In this study, 7.7% of the patients used medication that might act as a cofactor. Antacids and acid neutralizing medication were used in 5% of the patients, NSAID's in 2.2% and beta blockers, ACEIs and ARBs in ≤0.6%. This is lower than the use of this medication in the general Dutch population. In 2012 Dutch pharmacies delivered antacids to 15–20%, NSAID's to 20% and beta blockers and ACEIs together to 15–20% of the general Dutch population 27. It is probable that these results manifested as a result of the study population having a lower mean age than the Dutch population as a whole; 33 years versus 40–41 years 28. It is likely that the frequency of medication intake increases with age.

This study reported about the frequency with which patients use medication that might act as a cofactor in food allergy. Evidence about the role of this cofactor is scarce and there is discrepancy in outcomes. Only a study in mice showed that the use of proton‐pomp‐inhibitors increases the risk of anaphylaxis 29. Untersmayr et al. 30 suggested that in long‐term acid‐suppressed patients the anti‐ulcer treatment primes the development of IgE toward dietary compounds. Further, the CICBAA (French food allergy network) demonstrated that in 0.9–4.7% of the anaphylactic reactions to food, beta blockers played a role and ACEIs and ARBs in respectively 0–0.1% and 0.9–2.4% 10. In conclusion, based on available evidence, involvement of these types of medication might cause more severe food allergic reactions. However, additional studies are needed to confirm the relative contribution of these drugs to the severity of food allergic reactions. Given that these types of medication are used in 7.7% or more of food allergic patients, it is important that physicians inform patients about the potential influence on their food allergy and check this during follow‐up.

This study demonstrated that 13% of the patients reported to experience more severe allergic symptoms to food after involvement of a cofactor. In patients with severe food allergy, the frequency of involvement of cofactors was significantly higher than in patients with mild or moderate food allergy (16% vs. 10%). The most frequently involved cofactor was physical exercise. Other studies reported a higher frequency of cofactors in anaphylactic reactions to food, namely 26–39% in adults 10, 12 and 18.3% in a mixed population of children and adults 18. Since in our study two‐third of the patients indicated not to know if a cofactor influenced their allergic reaction, it can be assumed that patients might be largely unaware of the potential role of cofactors. In literature, it was earlier hypothesized that increased awareness is needed 7, 31. On the other hand, most patients in the age group of this study (mean age: 33 years) regularly consume alcohol and perform physical exercise 32, 33. We suppose that patients should have noticed it when these cofactors are associated with their allergic reactions, which confirms the low frequency. Another explanation for the lower frequency found, is that our study focused only on three cofactors which were regularly reported in literature, but not on other cofactors like infections, hormonal influence and body temperature 6, 7. Still, cofactors seem to be involved in 13% or more of the patients with food allergy. This makes cofactors important to take into account in diagnostic measures and doctors’ advice.

With respect to the frequency of involvement of specific cofactors, we demonstrated that 10% of the patients reported physical exercise as a cofactor, 5% alcohol intake and 0.6% intake of analgesics. There is wide range in the frequency of these three cofactors in literature. The frequency of physical exercise as a cofactor was earlier reported in a range of 0–15.9% of the anaphylactic reactions in adults 10, 12, 19. Wolbing et al. 6 reported that alcohol was a cofactor in anaphylaxis in 15.2% of the patients. Kanny et al. 9 demonstrated that 13% of the food allergic patients (children and adults) reported alcohol consumption as a cofactor. The CICBAA 10 showed that NSAIDs are a cofactor in 1.2–4.7% of the anaphylactic events. Cardona et al. 31 demonstrated that NSAIDs are involved in 58% of cofactor‐enhanced food allergic reactions. Kanny et al. 9 reported that alcohol or NSAID intake is significantly more frequent in anaphylactic shocks than in mild reactions to food. These differences may be caused by the differences in population and differences in method of data collection.

Physical exercise is a well‐known cofactor in food‐dependent exercise induced anaphylaxis (FDEIA). In FDEIA, physical exercise is a prerequisite to induce allergic reactions to food 6, 7. FDEIA is accepted as a separate clinical entity. Wheat is the most prevalent cause. Until now the precise mechanism is still unclear 13, 15, 34. It was demonstrated that patients with WDEAI often have IgE reacting to omega‐5 Gliadin and HMW‐Glutenin 15, 34, 35, 36. In our study only one patient had FDEIA, but not related to wheat but to chicken meat and hen's egg.

Our study demonstrated that some patients had more severe allergic reactions after alcohol consumption, which is confirmed by Wolbing et al. 6 and Niggeman et al. 7. In literature several underlying mechanisms are suggested for this cofactor 7, 16, 17. It was also shown that a high intake of alcohol is associated with increased total serum IgE levels and allergic sensitization 16, 17. Remarkably the effect was different for pollen (higher degree of sensitization) and house dust mite (lower degree of sensitization) 16. However the underlying mechanism is far from being understood 16, 17. We found that the use of alcohol was reported to enhance the severity of a food allergic reaction. Since alcohol was reported to result in relaxation of tight junctions in the gut epithelium, this might lead to increased allergen uptake and in turn to more severe reactions 6, 7. More studies are needed to understand the (probably different) pathophysiological mechanisms behind the alcohol as a cofactor.

It is known that allergic reactions could be more severe with unstable asthma and during the pollen season 37, 38. So, a combination of unstable atopic comorbidities and involvement of cofactors could lead to even more severe reactions. Since atopic comorbidities are frequently present in the food allergic population it seems important to minimize a potential negative influence by optimizing the treatment of any atopic comorbidity.

In this study many patients (67%) used medication (daily or on demand) that reduces severity of allergic symptoms, whereof antihistamines were most often used (56%). These factors 39, are important to consider as well. Notably, most literature so far only reported about factors that increased the severity of food allergy, but little attention was paid to factors that might decrease the severity of an allergic reaction, which might be at least as important.

This study gives information on the possible frequency in which cofactors might occur in the food allergic population. However, the study provides no information about a possible influence of cofactors on the minimal eliciting dose or individual thresholds of patients nor on the proportion of the food allergic population in which cofactors might influence thresholds or may play another role (not affecting thresholds). No clinical studies have been published yet that systematically studied the influence of cofactors on thresholds. However, in the total population of the present study the frequency of cofactors was only 13% and the frequency that patients use medication that are known as a potential cofactors was low, suggesting that cofactors, if influencing thresholds at all, probably will have limited influence on the dose‐distribution of minimum eliciting dose at a population level. Further research is needed to investigate the influence of cofactors on eliciting doses.

A limitation of this study was the self‐reported data and the possibility of recall bias and information bias. The diagnosis of food allergy and allergic rhino conjunctivitis was confirmed by the results of diagnostic tests (SPT, ImmunoCAP and food challenge). The criteria of Williams 25 were followed to diagnose atopic dermatitis. However, the criteria “Onset under the age of 2 years” was excluded because of missing data on this item. For the diagnosis of asthma the criteria of the GINA guidelines 23 were used. However some criteria were excluded because no data was collected about these items. This makes the diagnoses of atopic dermatitis and asthma somewhat less certain.

In conclusion, the results of this study show that only a small percentage of patients (7.7%) used medication that might aggravate food allergic reactions. Physical exercise and alcohol consumption were the most frequently reported cofactors associated with more severe allergic symptoms in patients with food allergy, but still in only 10% or less. Our results indicated that it is important to increase the awareness both among patients and health professionals.

## Author Contributions

A. Versluis, H. van Os‐Medendorp, and A.C. Knulst contributed to study design, data collection, data analysis and interpretation and writing the manuscript. A.G. Kruizinga, W.M. Blom, and G.F. Houben contributed to interpretation of results and manuscript revision. Each author listed on the manuscript has seen and approved the submission of this version of the manuscript and takes full responsibility for the manuscript.

## Conflicts of Interest

None declared.
